# Hospitalization, Asthma Phenotypes, and Readmission Rates in Pre-school Asthma

**DOI:** 10.3389/fped.2020.562843

**Published:** 2020-11-20

**Authors:** Helena Donath, Sven Kluge, Georgia Sideri, Jordis Trischler, Silivija P. Jerkic, Johannes Schulze, Stefan Zielen, Katharina Blumchen

**Affiliations:** Division of Allergology, Pulmonology and Cystic Fibrosis, Department for Children and Adolescents, Goethe University, Frankfurt, Germany

**Keywords:** hospitalization, readmission rates, tailored treatment schedule, asthma phenotypes, pre-school asthma

## Abstract

**Objective:** Children with pre-school asthma suffer disproportionally more often from severe asthma exacerbations with emergency visits and hospital admissions compared to school children. Despite this high disease burden, there are only a few reports looking at this particular severe asthma cohort. Similarly, there is little real-life research on the distribution of asthma phenotypes and personalized treatment at discharge in this age group.

**Patients and Methods:** Retrospective analysis of the electronic charts of all children aged 1–5 years with asthma hospitalizations (ICD J45) at the Frankfurt University between 2008 and 2017. An acute severe asthma exacerbation was defined as dyspnea, oxygen demand, and/or systemic steroid therapy. Age, gender, duration of hospitalization, asthma phenotype, treatment, and readmission rate were analyzed.

**Results:** Of 572 patients, 205 met the definition of acute severe asthma. The phenotypic characterization showed 56.1% had allergic asthma, 15.2% eosinophilic asthma and 28.7% non-allergic asthma. Of these patients, 71.7% were discharged with inhaled corticosteroids (ICS) or ICS + long-acting-beta-agonists (LABA), 15.1% with leukotriene antagonists (LTRA) and 7.3% salbutamol on demand. The rate of emergency presentations (emergency department and readmission) within 12 months after discharge was high (*n* = 42; 20.5%). No phenotype tailored treatment was detectable. Neither the number of eosinophils (>300/μl) nor the treatment at discharge had an effect on emergency visits and readmission rate.

**Conclusion:** Despite protective therapy with ICS, ICS + LABA, or LTRA, the readmission rate was high. Thus, current care and treatment strategies should be reevaluated continuously, in order to better control asthma in pre-school children and prevent hospitalization.

## Introduction

In primary care and emergency departments, acute asthma exacerbations are one of the main causes for pediatric emergency visits (1, 2). Although the prevalence of asthma among school children is higher than among pre-school children, severe asthma exacerbations with emergency consultations and hospitalizations are much more common in pre-school children (1, 3, 4). In addition, mortality from pre-school asthma is very high (4). Most likely, due to smaller airways and possibly increased bronchial hyperresponsiveness, pre-school children with asthma are more susceptible to severe asthma exacerbations compared to older children (5). Shortness of breath due to infections can quickly become life-threatening (3, 4). This explains the relatively high rate of emergency consultations and hospital admissions in pre-school children.

Early identification of children with pre-school asthma who are at risk for persistent asthma is an ongoing challenge for pediatricians. Approximately one-third of infants who wheeze in their first 3 years of life suffer from asthma related events such as wheezing and shortness of breath at school age (6). Thus, even mild wheezing episodes early in life may be a significant risk factor for persistent wheezing and asthma later in life (7, 8).

Early intervention with anti-inflammatory agents is indicated in pre-school asthma to prevent the development of uncontrolled asthma and frequent exacerbations, which lead to high health care utilization (9–11). Although studies are limited, data suggest that an asthma-like inflammation (presence of eosinophils and allergic sensitization) may be present at a very early age in some children with recurrent wheeze (11). Analyzing the heterogeneous group of pre-school asthma, the following phenotypes have been characterized (12–14): asthma with sensitization defined by specific IgE (sIgE) > 0.7 KU/L and/or a positive skin prick-test to common allergens, eosinophilic asthma with elevated blood eosinophils (>300 eosinophils/μl) without presence of sensitization, and non-allergic asthma without TH2- inflammation. It is important to note that approximately half of the pre-school asthma patients do not present with TH2- inflammation, and may show a TH1- like neutrophilic airway inflammation as it was described in adults (15, 16).

Currently available anti-inflammatory treatments for first line therapy in pre-school asthma include inhaled corticosteroids (ICS) and the leukotriene receptor antagonist (LTRA) montelukast. Both, ICS, and LTRA, have been found to be effective in this age group (13, 17, 18). Biomarkers of TH2- inflammation like eosinophils, total and elevated sIgE sensitization are useful in the prediction of future exacerbations and may identify children who are likely to respond favorably to daily ICS treatment (13, 19). However, ICS have been shown to reduce severe exacerbations effectively by only 44% in pre-school asthma patients (13, 20). Thus, asthma exacerbations still occur frequently despite the regular use of ICS. Even quintupling the ICS dose did not reduce the rate of severe asthma exacerbations and may be associated with diminished linear growth (21, 22). In addition, in children with persistent asthma, the combination of ICS and additional long acting beta agonists (LABA) was not associated with a significant reduction of exacerbations requiring systemic steroids (23, 24).

Severe asthma exacerbations with hospitalization are a pivotal event that requires careful reevaluation of the previous asthma treatment and a new personalized approach of future asthma management (1, 2, 13). Ideally, the current treatment options should be based on the underlying asthma phenotype (13). Therefore, the aim of this retrospective real-life study was to carry out a detailed analysis of hospitalized pre-school children with acute severe asthma exacerbations (duration of hospitalization, age, gender, therapy before admission and after discharge, the asthma phenotype and the readmission rate within 12 months). In addition, hurdles of physicians' decision to base the current asthma management on the underlying phenotype at discharge as well as the readmission rate in regard to the phenotype were investigated.

## Methods

We carried out a retrospective analysis of the electronic medical records (Orbis, version 08.04.33, Orbis AG, Saarbrücken, Germany) of all asthma admissions of patients aged 1–5 years between January 2008 and December 2017 at the University Hospital in Frankfurt using ICD-10 code “J45” to identify children who have had an acute severe asthma exacerbation. Children suffering from congenital heart disease, genetic diseases such as cystic fibrosis or complex syndromes affecting the respiratory system, or other significant lung diseases such as bronchopulmonary dysplasia and immunodeficiencies were excluded.

Since the differentiation between acute viral bronchiolitis/pneumonia and asthma is very difficult the criteria for diagnosing asthma were defined as follows: At least two physician documented episodes of wheezing in the last 12 months with demand of ß2-Mimetics (Salbutamol), inhaled corticosteroids (ICS), or systemic corticosteroids at 3 consecutive days before hospitalization. Thus, a minimum of three episodes of wheeze were documented in all patients diagnosed as asthma. Minor but secondary criteria of diagnosing asthma were bronchodilator responsiveness during wheeze, family history, atopic dermatitis, and presence of allergy. In addition, an acute asthma exacerbation was defined as acute dyspnea, tachypnea, oxygen demand, and/or systemic steroid therapy during hospitalization.

All patients fulfilling the mentioned criteria were diagnosed with asthma and advised to get regular appointments in our outpatient asthma clinic. These patients were screened for emergency visits and readmission to our hospital during the following 12 months after the first hospitalization due to severe acute asthma exacerbation.

Measurement of compliance: Asthma treatment with preventers like ICS, ICS + LABA, and LTRA were documented at first hospitalization and discharge from hospital and again at subsequent emergency visits/readmission within 12 months after discharge of the first hospitalization. Therefore, compliance/treatment adherence was determined in all patients with readmission/emergency visit.

### Outcome Parameters

Days of hospitalization at first admission, oxygen demand based on oxygen saturation, blood eosinophil counts, total Immunoglobulin E (IgE), sIgE, and patient's treatment at first admission were evaluated as outcome parameters.

Asthma phenotypes were defined as described (14, 20):

1. **Allergic asthma with sensitization** defined by sIgE ≥0.75 KU/L and/or a positive skin prick-test ≥3 mm to common allergens like birch, grass, mites, molds, cat, dog, horse, milk, and egg.
2. **Eosinophilic asthma without sensitization** defined by elevated eosinophils > 300 μl without presence of sIgE and/or a positive skin prick-test.
3. **Non-allergic, non-eosinophilic asthma** without elevated eosinophils > 300 μl and without sensitization.
4. **Asthma with atopic dermatitis:** This phenotype was defined clinically by the presence of eczema. In addition, sensitization was defined either by elevated sIgE ≥0.75 K/UL and/or a positive skin prick-test by a wheal ≥3 mm. Since patients with atopic dermatitis and asthma often show sensitization and elevated eosinophils, there may be some overlap between patient group 1 and 2.

### Statistics

The study was a retrospective study, and only descriptive results are presented. Basic descriptive statistics including absolute and relative frequency distributions are reported. Statistical analysis was performed with GraphPad Prism 5.0 (GraphPad Software, San Diego, CA, USA). Clinical characteristics (age, sex, treatment, eosinophils, IgE, sIgE, sensitization, and phenotypes) of patients with and without readmission were compared by the Mann–Whitney U-test or the Wilcoxon–Mann–Whitney-test. Ethics approval was obtained from the ethics committee of the Goethe University in Frankfurt (Application Number 427/19).

The primary endpoint was readmission rate/emergency visits (number of patients) within 12 months with and without asthma treatment.

Secondary parameters:

1. Proportion (%) of patients with readmission (<30 days, <90 days, and up to 12 months) and asthma treatment.
2. Underlying asthma phenotype and readmission.
3. Analysis of asthma therapy (ICS, ICS + LABA, LTRA, vs. no therapy) before and after readmission according to phenotype.
4. Compliance of patients at readmission.

Exploratory parameter:

Readmission rate in patients with significant eosinophilia >300/μl vs. normal eosinophil count.

## Results

### Description of the Acute Asthma Cohort

**Figure 1:**
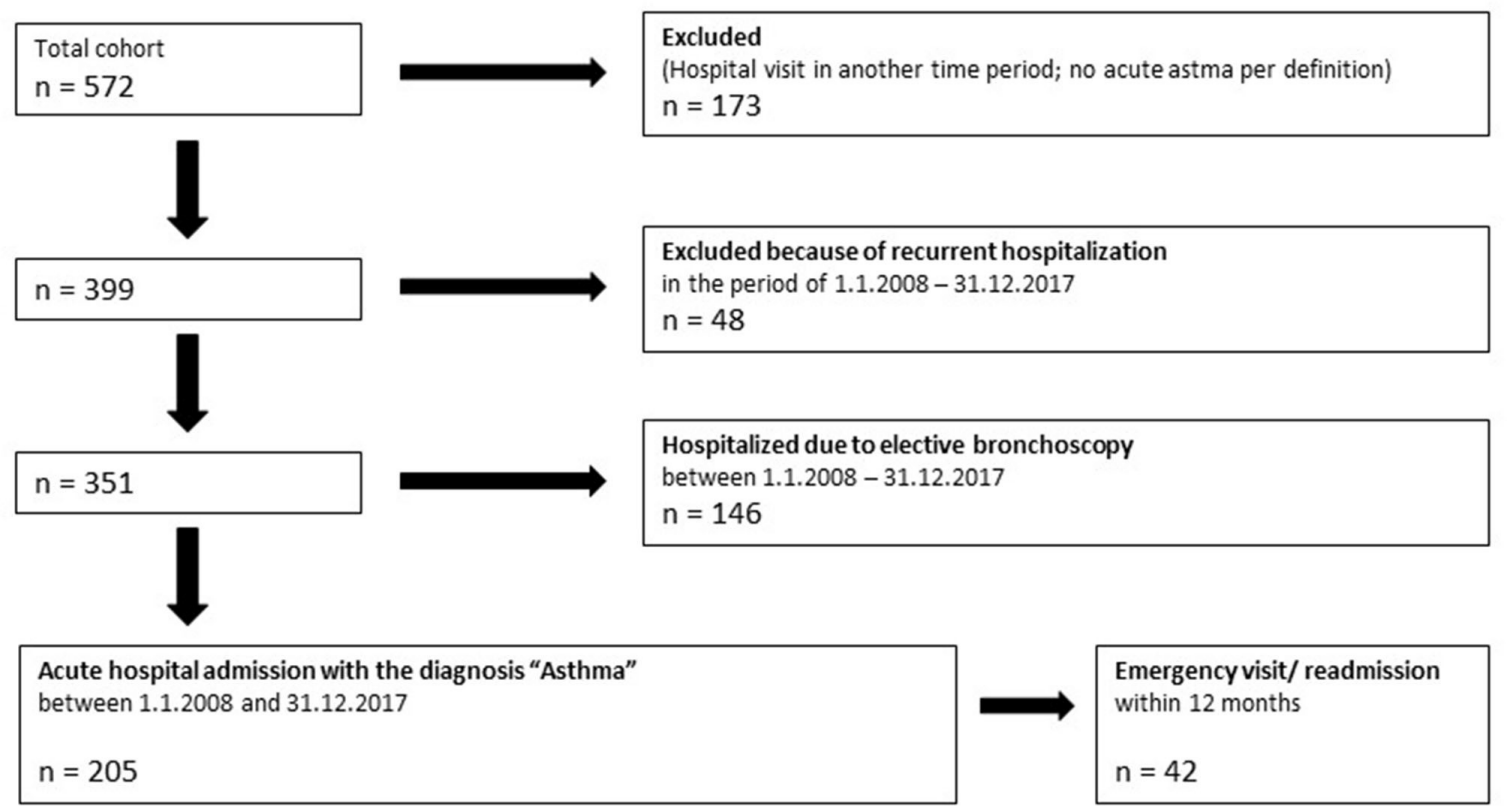
Flowchart of study patients.

In 2008–2017, 572 patients were hospitalized with the diagnosis of acute asthma exacerbation (ICD-10 code: J45). Of these patients, 205 met the definition of acute severe asthma (Figure 1). Most patients were treated with systemic steroids (84.9%) and received oxygen (56.1%). The majority of patients were male (72.68%) (Table 1).

**Table 1:** Characteristics of all patients and patients with emergency visits/readmission.

**Demographic data at hospitalization**	**All patients (*n* = 205)**	**Emergency visits/readmission (*n* = 42)**
Age, months (range)	44 (12–71)	41 (12–69)
Sex (male/female), *n*	149/56	30/12
Atopic dermatitis, %	9.3	11.9
Number of patients with an oxygen saturation <94, %	39.5	42.9
Number of patients with oxygen demand %	56.1	61.9
Systemic steroids at admission, %	84.9	90.5
**Treatment before admission**, ***n*****/%**	119 (58.0)	26 (61.9)
No daily treatment, *n* (%)	86 (42.0)	16 (38.1)
Salbutamol on demand, *n* (%)	57 (27.8)	11 (26.2)
ICS, *n* (%)	30 (14.6)	7 (16.7)
ICS + LABA, *n* (%)	25 (12.2)	6 (14.39)
LTRA, *n* (%)	7 (3.49)	2 (4.79)
**Duration of hospital stay (days)**	3 ± 2.71	3 ± 1.68
**Treatment at discharge**, ***n*** **(%)**	193 (94.1)	42 (100.0)
No daily treatment, *n* (%)	12 (85.9)	0 (0.09)
Salbutamol on demand, *n* (%)	15 (7.3)	6 (14.3)
ICS, % *n* (%)	90 (43.9)	15 (35.7)
ICS + LABA, *n* (%)	57 (27.8)	13 (31.0)
LTRA, *n* (%)	31 (15.1)	8 (19.0)

*Median and Range; ICS, inhaled corticosteroid; LABA, long-acting β_2_-agonist; LTRA, leukotriene receptor antagonist*.

At first hospitalization, 42% of patients had no treatment, 27.8% were treated with salbutamol on demand, and only 62 (30.3%) patients received treatment with ICS, ICS + LABA, or LTRA (Table 1). The clinical characteristics of patients with and without treatment were similar (data not shown).

**Figure 2:**
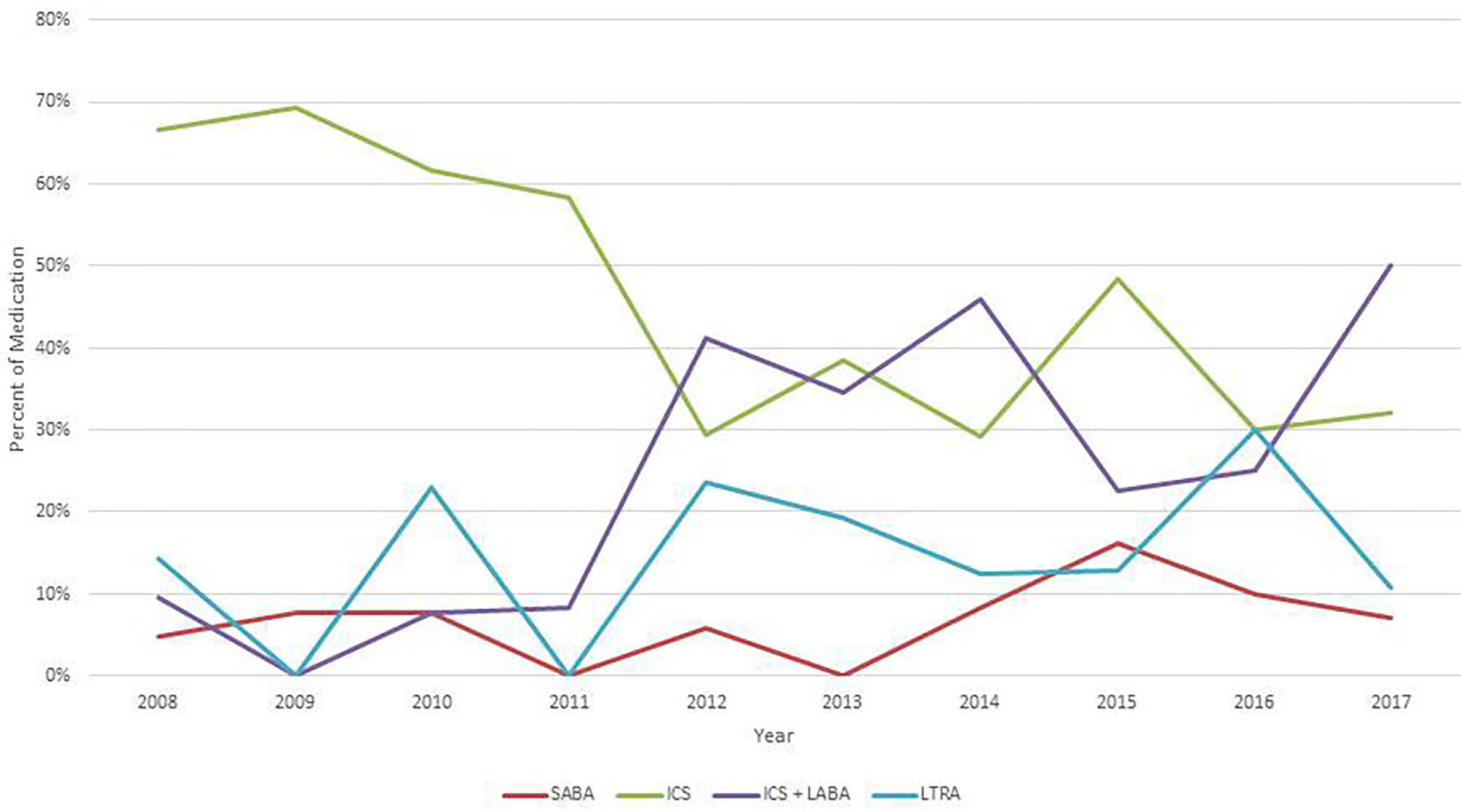
Medication at discharge 2008–2017. ICS, inhaled corticosteroids; LABA, long-acting β2-agonists; SABA, short-acting β2-agonists; LTRA, leukotriene receptor antagonist.

At discharge of the first hospitalization due to the severe acute asthma exacerbation, 86.8% of the patients were treated with daily medication; 43.9% patients received ICS, 27.8% ICS + LABA and 15.1% LTRA (Table 1). Treatment at discharge showed some variation during the 10 years of observation (Figure 2). ICS was the leading asthma control therapy from 2008 to 2011. From 2011, the number of patients who received a combination of ICS + LABA was significantly increased while there was a simultaneous decrease of monotherapy with ICS.

### Readmission/Emergency Visits Within 12 Months

The number of emergency presentation (emergency department and or re-hospitalization) within 12 months was high (*n* = 42; 20.5%). The clinical characteristics of these patients are shown in Table 1. Of 42 patients with readmission/emergency visits, 6 were treated with salbutamol on demand, whereas 36 received protective daily treatment. Despite recommended daily treatment, 16 of 42 patients showed a severe exacerbation within 90 days (Table 2). The recommended treatment of these 16 patients is shown in Table 2: ICS 5 of 15 (33.6%); ICS + LABA 7 of 14 (50.0%) and LTRA 4 of 8 (50%). Twenty-five patients had severe exacerbations with readmission/emergency visits within 90 and 360 days. Next, we analyzed the relationship between the age of the children and readmission/emergency visits. Of 29 children aged 1–2 years, 12 children (47.3%) had an emergency visit/readmission, whereas of 45 children aged 5–6 years only 7 (15.6%) had an emergency visit/readmission.

**Table 2:** Emergency visits/readmission and compliance.

	**LTRA**	**ICS**	**ICS + LABA**	**Emergency visits/readmission^**#**^**	**Compliance total (%)**
Total at discharge *n*	8	15	13	42	100
Exacerbation within 30 days with/and without treatment (*n*)	3/0	2/0	1/0	7*	100
Exacerbation within 90 days with/and without treatment (*n*)	1/0	3/0	3/3	10	70.0
Exacerbation after 90–360 days with/and without treatment (*n*)	0/4	4/6	2/4	25^*^	30.0

# *Number of patients*;

* *Six patients were discharged with Salbutamol on demand, of these patients one exacerbated within 30 days, and five after 90–360 days*.

In the next step, we analyzed patient compliance. Compliance decreased continuously depending on the duration of time between the inpatient stay and the emergency presentation (Table 2). Most of the patients were taking their long-term controller therapy within 90 days when presented, while compliance decreased to 30% within 12 months (Table 2).

### Asthma Phenotype

**Figure 3:**
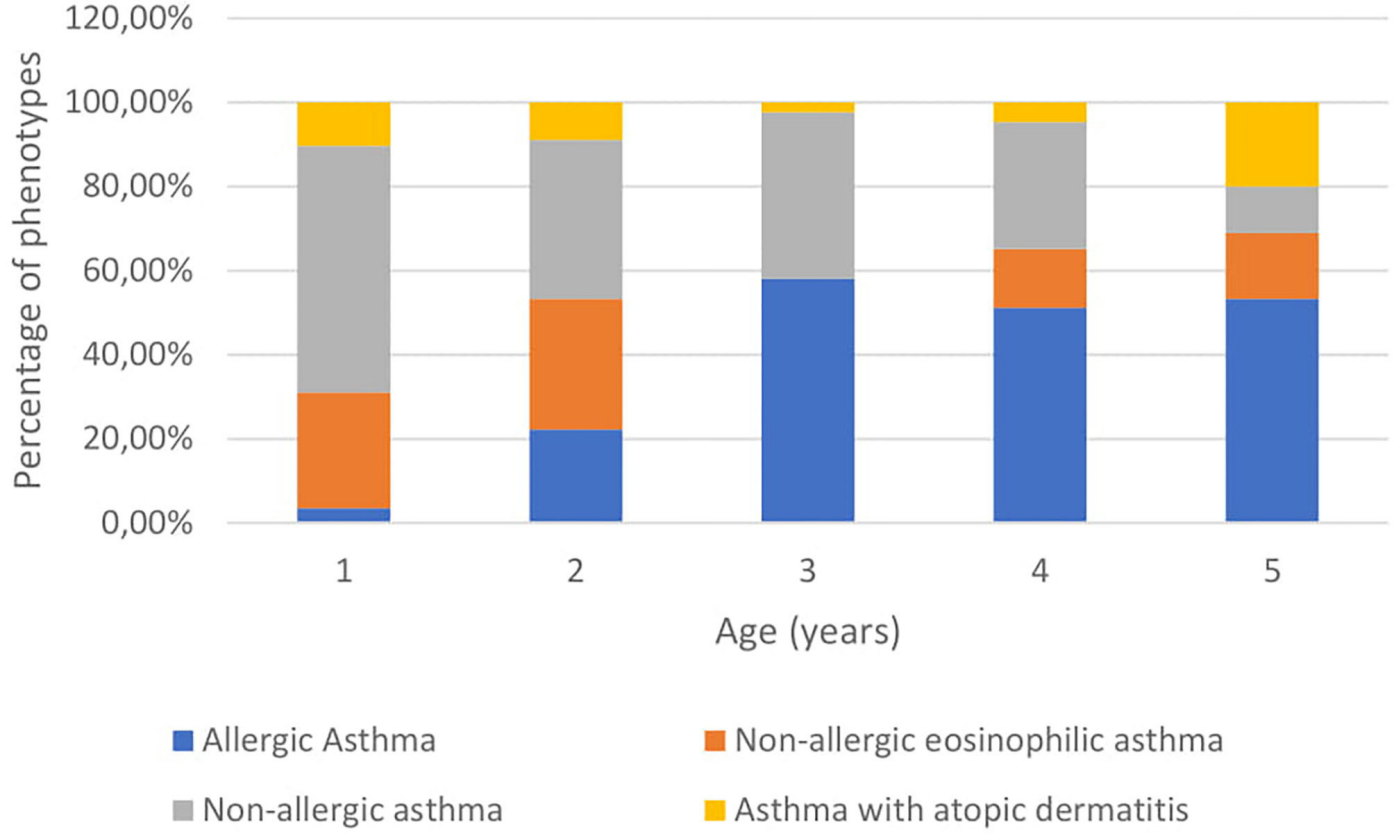
Distribution of phenotypes according to age.

In this real-life study, determination of the asthma phenotypes were possible in 171 (83.9%) of 205 patients. The phenotype distribution of allergic asthma with sensitization increased with age, whereas the number of non-allergic, non-eosinophilic asthma decreased (Figure 3). In addition, measurement of sIgE was done in 80 (39%) of 205 patients.

The percentage of patients with the phenotype allergic asthma with sensitization was similar distributed when percentage was based on skin prick-test (*n* = 172; 56%) or sIgE (*n* = 80; 60%); eosinophilic asthma without sensitization based on prick-test 15.2% vs. based on sIgE 11.25% and non-allergic, non-eosinophilic asthma 28.7 vs. 28.75%. Since patients with asthma and atopic dermatitis may represent a distinct phenotype we analyzed the phenotype distribution in patients with sIgE measurements in detail (Table 3). Patients with allergic asthma had significantly higher levels of eosinophils (*p* ≤ 0.01) and total IgE (*p* ≤ 0.05) than patients with non-allergic, non-eosinophilic asthma. As shown the number of readmissions were higher in patients with asthma and atopic dermatitis (25%) and patients with non-allergic, non-eosinophilic asthma (28.6%) compared to allergic asthma (10.3%) and eosinophilic asthma (0%). However, due to the small numbers of patients this result was not significant (Table 3).

**Table 3:** Characteristics of patients with distinct phenotypes.

**Demographic data at hospitalization**	**Allergic patients *n* = 39**	**Eosinophilic asthma *n* = 8**	**Non-allergic asthma *n* = 21**	**Asthma with atopic dermatitis *n* = 12**
Age, months (range)	51 ± 13.96	48.5 ± 17.17	37 ± 17.87	36.5 ± 19.70
Sex (male), *n* (%)	31 (79.5)	7 (87.5)	13 (61.9)	9 (75.0)
Duration of hospital stay (days)	3 ± 1.32	2.5 ± 1.58	3 ± 2.02	3 ± 1.16
Eosinophils (μl)	300^*^	400	85	210
	10–1,390	310–800	10–270	20–1,100
Total IgE (KU/L)	389^*^	51	45	507
	72–11,250	18–305	2–494	9–7,732
Total IgE >100, *n* (%)	19 (48.7)	2 (25.0)	6 (28.6)	8 (66.7)
**Treatment before admission**, ***n*** **(%)**	22 (56.4)	2 (25.0)	11 (52.4)	5 (41.7)
No treatment, *n* (%)	17 (43.6)	6 (75.0)	10 (47.6)	7 (58.3)
Salbutamol, *n* (%)	15 (38.5)	2 (25.0)	5 (23.8)	3 (25.0)
ICS, *n* (%)	4 (10.3)	0 (0.0)	4 (19.0)	0 (0.0)
ICS + LABA, *n* (%)	3 (7.7)	0 (0.0)	1 (4.8)	0 (0.0)
LTRA, *n* (%)	0 (0.0)	0 (0.0)	1 (4.8)	2 (16.7)
**Treatment at discharge**, ***n*** **(%)**	37 (94.8)	8 (100.0)	19 (90.5)	11 (91.7)
No treatment, *n* (%)	2 (5.2)	0 (0.0)	2 (9.5)	1 (8.3)
Salbutamol, *n* (%)	5 (12.8)	1 (12.5)	0 (0.0)	1 (8.3)
ICS, *n* (%)	9 (23.1)	2 (25.0)	8 (38.1)	4 (33.3)
ICS + LABA, *n* (%)	16 (41.0)	2 (25.0)	5 (23.8)	1 (8.3)
LTRA, *n* (%)	7 (17.9)	3 (37.5)	6 (28.6)	5 (41.7)
Emergency visits [*n* (%)]	4 (10.3)	0 (0.0)	2 (9.5)	3 (25.0)
Readmission [*n* (%)]	4 (10.3)	0 (0.0)	6 (28.6)	3 (25.0)

*Median and Range; ICS, inhaled corticosteroid; LABA, long-acting β_2_-agonist; LTRA, leukotriene receptor antagonist*.

* *Patients with allergic asthma had significant higher eosinophils (p < 0.01) and total IgE (p < 0.05) compared to patients with non-allergic asthma*.

Controller treatment of patients with different phenotypes was similar at discharge (Table 3). No phenotype tailored treatment was detectable. Neither the number of eosinophils (>300/μl) nor did the treatment at discharge have an effect on emergency visits and readmission rate (Table 3).

## Discussion

In the 1–5-year-old age group, severe asthma exacerbations with emergency visits and hospitalization are very common (1, 3). Admissions related to asthma represent a large proportion of pediatric health-care resources and account for a substantial amount of health-care expenditures (1, 25, 26). The results of this study show that current treatment strategies are not sufficient to control patients with severe pre-school asthma adequately. At discharge, 85.8% of patients were advised to use daily anti-inflammatory treatment with inhaled ICS, ICS + LABA, or LTRA. However, 20.5% of these patients experienced a severe episode with an emergency visit or re-hospitalization within 12 months. The high rate for re-hospitalization of virally induced asthma is well-known and has been shown by other studies (20, 27). In our study, younger children contribute significantly to the high readmission rate. Younger children have increased vulnerability to viral infections due to small airways and possibly increased bronchial airway reactivity, in comparison with older children. Respiratory distress in the setting of viral infection can rapidly become life-threatening. For this reason, the relatively high utilization of hospital admissions in pre-schoolers aged 1–2 years may be well-explained. Readmissions for pediatric asthma are a significant public health problem (25–27). Furthermore, it is well-known that a recent severe asthma exacerbation increases the risk of subsequent exacerbation substantially (28).

The number of readmissions in our cohort with acute severe asthma within 12 months was high despite potent asthma treatment at discharge. There is some evidence that the period of more than 30 days between an initial hospitalization and readmission for severe asthma is more likely to reflect modifiable factors such as asthma management upon discharge (29). However, treatment with ICS or ICS + LABA was only partially effective in the first 90 days after discharge, whereas at longer follow-up, the adherence to recommended treatment was poor. Whether this was due to poor advice, fear to side-effects or limited excess to health utilization remains speculative. In addition, since treatment adherence was not measured by objective methods like electronic devices and pick-up/refill rates, this question can only be addressed adequately in a prospective trial.

The annual exacerbation rate of our patients was rather constant during the past 10 years although there was a significant treatment switch of our hospital policy from ICS to an ICS + LABA combination, which was successfully implemented as set up treatment in uncontrolled asthma of school children (30) (Figure 2). Although the number of patients with adherence to treatment was small, our data are in accordance with two recent reports which showed that the ICS + LABA combination was not superior to ICS alone in pre-school asthma (23, 24).

Identifying children at high risk for frequent severe exacerbations may significantly reduce repeated hospitalizations in childhood asthma. It has been suggested that a more personalized treatment considering distinct wheezing phenotypes is more efficient and may prevent frequent exacerbations (13, 19, 20). However, defining these wheezing phenotypes in young children in a real-world situation is quite difficult. The changeability in disease phenotype and heterogeneity of the patient population make it difficult to classify pre-school patients with asthma into a distinct phenotype (14). So far, most studies have described phenotypes in birth cohorts to define their long-term outcome, whereas only few studies have analyzed patients with acute severe pre-school asthma. This is the strength of our study which analyzed a severe asthma cohort at a given time point during hospitalization. The majority of classified patients, 60.0%, had a TH2- phenotype with high total and sIgE. The so-called non-allergic, non-eosinophilic asthma group with absence of a TH2- phenotype was present in 28.75% of patients and patients with pure eosinophilic asthma were only characterized in 11.25%. The number of emergency visits and readmissions were higher in patients with asthma and atopic dermatitis (25%) and non-allergic, non-eosinophilic asthma (28.6%) compared to the allergic phenotype. It can be discussed why asthma with atopic dermatitis is defined as a separate phenotype, however, since atopic dermatitis with sensitization is a risk factor for persistent asthma later in life, we consider it to be a distinct phenotype (27). Nonetheless, the number of patients with exacerbations was too small to be significant. Interestingly, treatment at discharge was independent from the underlying asthma phenotype. It has to be discussed why ICS or ICS + LABA combinations were not more commonly prescribed in the allergic asthma group since a favorable treatment response has been reported by several studies (13, 19, 20). On the contrary, in the non-allergic group either mono LTRA or a combination of LTRA and ICS or even azithromycin is the recommended treatment (13). Possibly, the phenotype-based concept was not well-implemented in our hospital or the current guideline that ICS is the best choice in any asthma phenotype is still believed and trained in Germany. Although some risk factors for asthma in pre-school children have been well-described such as presence of allergy, eosinophilia or eczema, most decisions rely solely on the individual judgment and expertise of the treating physician (13, 19, 20, 31, 32).

On the other hand, validated treatment options are scarce due to paucity of data overall and lack of conclusive studies in such a young patient population. Global Initiative for Asthma (GINA) recommends daily treatment with low-dose ICS for pre-school children with persistent asthmatic symptoms who develop a pattern that becomes consistent with asthma (10). If symptoms do not improve or become worse, increasing the dose of ICS or adding additional treatment with LTRA is recommended (10, 13). However, ICS therapy in pre-school children with persistent asthma has its limitations: whilst a recent meta-analysis found strong evidence to support daily ICS use, with a reduction in exacerbations of 44%, total prevention of exacerbations was not achieved (20).

Optimizing asthma management among pre-school children is an unmet need, since this age group suffers from significant morbidity, including higher rates of hospitalization and intensive care unit admission (1, 3, 4). A new treatment option is tiotropium, which was found to be a well-tolerated and efficacious add-on therapy to ICS plus one or more controller medications in several clinical trials in children and adolescents (33–36). Two recent trials in pre-school children showed that the addition of tiotropium to ICS was well-tolerated and had the potential to reduce the risk of asthma-related events and severe asthma exacerbations with need of systemic steroids (35, 36). However, tiotropium is not licensed as add on therapy in this age group so far.

Our study has several limitations; these include the fact that it was a retrospective analysis and not a prospective or randomized clinical trial. The endpoints for this study were also exploratory and used for descriptive statistical analyses only. In addition, many patients were treated at discharge with an ICS + LABA combination and not with an ICS mono treatment as recommended by current guidelines; this limits the generalizability of our study. Further, no follow-up of the patients was done; we cannot be sure that the diagnosis of asthma was definite, because in the age group 1–2 years, some patients may suffer from bronchiolitis rather than asthma.

## Conclusion

Hospital readmissions due to severe asthma are more frequent in the pediatric pre-school population than in the school age. In order to identify patients at high risk for readmission, we retrospectively analyzed the medical records and examined the population according to age, sex, length of hospitalization, phenotype of asthma, and type of therapy. Although we could not identify typical demographics or clinical characteristics to predict the individual exacerbation risk, we demonstrated that phenotype-based treatment concepts are not yet realized in real-life care. The readmission rate in pre-school asthma is still high and current treatment strategies are not sufficient to adequately control pre-school patients with severe asthma.

## Research in Context

- Evidence before this study
  We reviewed literature on PubMed before writing this manuscript (April 2020) for recent publications on severe asthma in pre-school children. Despite this high disease burden, there are only a few detailed reports on hospitalization and readmission of this particularly severe asthma cohort.
- Added value of this study
  This retrospective analysis provides additional evidence that severe exacerbations are a frequent finding in pre-school asthma and that readmission rates are high. No phenotype tailored treatment was detectable. Neither the number of eosinophils (>300/μl) nor the treatment at discharge had an effect on emergency visits and readmission rate.
- Implications of all the available evidence
  Despite protective therapy with ICS, ICS + LABA, or LTRA, the readmission rate was high. Current care and treatment are not sufficient to adequately control severe asthma in pre-school children.

## Data Availability Statement

The raw data supporting the conclusions of this article will be made available by the authors, without undue reservation.

## Ethics Statement

Ethics approval was obtained from the Ethics Committee of the Goethe University in Frankfurt (Application Number 427/19).

## Author Contributions

SZ had the idea for the study. HD, SK, JT, SJ, SZ, and KB designed the study. SK and SZ obtained data. SK and SZ did data management. HD, SK, SZ, and KB did quality control, post-processing, and analysis. All authors contributed to writing, interpretation, critical review of data, and approved the final version.

## Conflict of Interest

SZ received a grant of 25.000 € of Boehringer Ingelheim for the current study. In addition, SZ reports grants and personal fees from bene-Arzneimittel GmbH, grants from ALK Arzneimittel, personal fees from Novartis GmbH, Boehringer Ingelheim, Lofarma GmbH, IMS HEALTH GmbH & Co. OHG, GSK, Stallergen, Procter and Gamble, Allergopharma GmbH, AstraZeneca, Sanofi/Pasteur, and Aimmune outside the submitted work. JS reports grants and personal fees from Novartis Pharma. KB reports grants and personal fees from Aimmune Therapeutics, personal fees from HAL Allergy, non-financial support from NOVARTIS, personal fees from Bencard Allergie, outside the submitted work. The remaining authors declare that the research was conducted in the absence of any commercial or financial relationships that could be construed as a potential conflict of interest.

## Abbreviations

sIgE: Specific Immunoglubulin E
ICS: Inhaled corticosteroids
LTRA: Leukotriene receptor antagonist
LABA: Long acting beta agonist
IgE: Immunoglobulin E
ICD: International statistical classification of diseases
GINA: Global initiative for asthma.
