# Supplementary material for: Hospitalization, Asthma Phenotypes, and Readmission Rates in Pre-school Asthma
Source: Front Pediatr. 2020 Nov 20;8:562843. doi: 10.3389/fped.2020.562843 (PMC7716437; doi:10.3389/fped.2020.562843)
Supplement: Supplementary file 1 [file Table_1.docx]

**Table supplement: Age distribution of 205 patients with pre-school asthma**

| Age of patients (years) | Percentage of 205 Patients with asthma |
| --- | --- |
| 1 -2 | **14.15** (n=29) |
| 2 -3 | **19.51** (n= 40) |
| 3-4 | **23.41** (n=48) |
| 4-5 | **20.98** (n=43) |
| 5-6 | **21,95** (n=45) |
